# Supplementary material for: Conditional chemoconnectomics (cCCTomics) as a strategy for efficient and conditional targeting of chemical transmission
Source: eLife. 2024 Apr 30;12:RP91927. doi: 10.7554/eLife.91927 (PMC11060718; doi:10.7554/eLife.91927)
Supplement: Figure 3—source data 3. [file elife-91927-fig3-data3.zip › Figure 3 source data3 part 2.zip!/Figure 3-Source data 3-part2/1053f3-1-CRISPResso_Report_t6djfp6u.zip!/CRISPResso_on_1053f3_4/1c.Alignment_barplot.pdf]

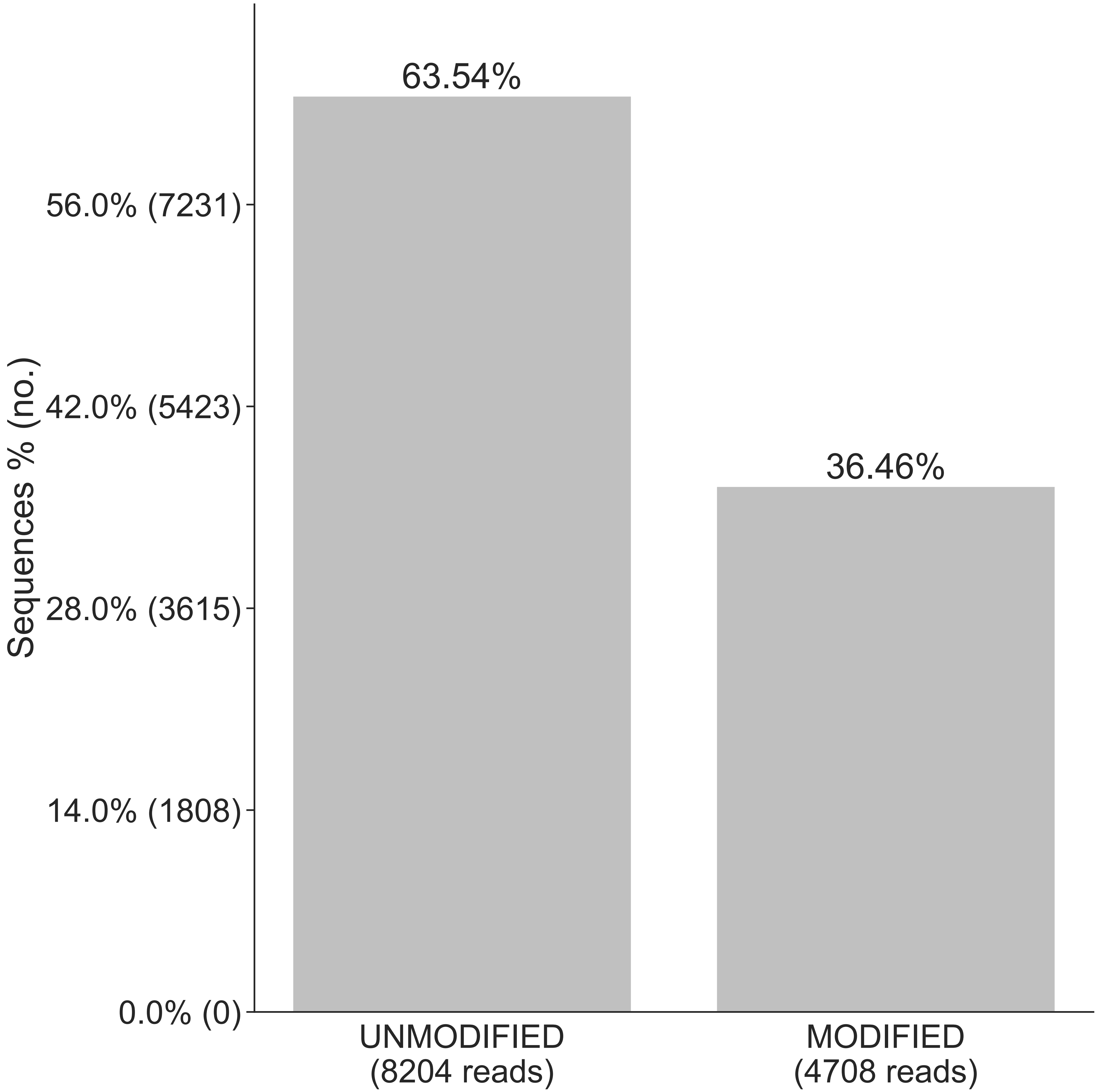

Sequences % (no.)
56.0% (7231)
42.0% (5423)
28.0% (3615)
14.0% (1808)
0.0% (0)
63.54%
36.46%
UNMODIFIED
(8204 reads)
MODIFIED
(4708 reads)
